# M2OR: a database of olfactory receptor–odorant pairs for understanding the molecular mechanisms of olfaction

**DOI:** 10.1093/nar/gkad886

**Published:** 2023-10-23

**Authors:** Maxence Lalis, Matej Hladiš, Samar Abi Khalil, Loïc Briand, Sébastien Fiorucci, Jérémie Topin

**Affiliations:** Institut de Chimie de Nice, Université Côte d’Azur, UMR 7272 CNRS, 06108 Nice, France; Institut de Chimie de Nice, Université Côte d’Azur, UMR 7272 CNRS, 06108 Nice, France; Institut de Chimie de Nice, Université Côte d’Azur, UMR 7272 CNRS, 06108 Nice, France; Centre des Sciences du Goût et de l’Alimentation, CNRS, INRAE, Institut Agro, Université de Bourgogne, F-21000 Dijon, France; Institut de Chimie de Nice, Université Côte d’Azur, UMR 7272 CNRS, 06108 Nice, France; Institut de Chimie de Nice, Université Côte d’Azur, UMR 7272 CNRS, 06108 Nice, France

## Abstract

Mammalian sense of smell is triggered by interaction between odorant molecules and a class of proteins, called olfactory receptors (ORs). These receptors, expressed at the surface of olfactory sensory neurons, encode myriad of distinct odors via a sophisticated activation pattern. However, determining the molecular recognition spectrum of ORs remains a major challenge. The Molecule to Olfactory Receptor database (M2OR, https://m2or.chemsensim.fr/) provides curated data that allows an easy exploration of the current state of the research on OR-molecule interaction. We have gathered a database of 75,050 bioassay experiments for 51 395 distinct OR-molecule pairs. Drawn from published literature and public databases, M2OR contains information about OR responses to molecules and their mixtures, receptor sequences and experimental details. Users can obtain information on the activity of a chosen molecule or a group of molecules, or search for agonists for a specific OR or a group of ORs. Advanced search allows for fine-grained queries using various metadata such as species or experimental assay system, and the database can be queried by multiple inputs via a batch search. Finally, for a given search query, users can access and download a curated aggregation of the experimental data into a binarized combinatorial code of olfaction.

## Graphical Abstract

**Graphical Abstract:**
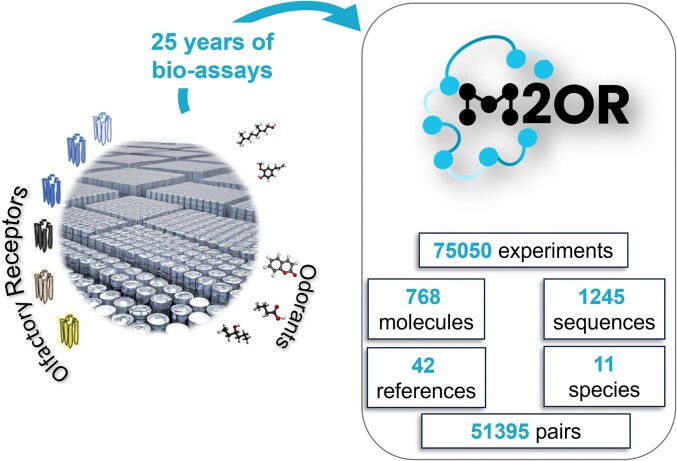

## Introduction

Mammalian sense of smell is based on a sophisticated mechanism for detection of volatile molecules by olfactory receptors (ORs) expressed in the membrane of sensory neurons (1). The first OR gene was discovered in 1991 (2) and since then, a thorough investigation has been carried out for various species including humans (3–7), mice (8), rats (9) and elephants (10). The total number of OR genes has been estimated to be approximately 400 in humans (11) and can reach nearly 5000 in elephants (10), making ORs the largest family of G-protein coupled receptors (GPCR).

Odorants are recognized by ORs according to a combinatorial code: a molecule can activate a subset of receptors and each receptor can respond to several different molecules (12). Understanding the mechanism by which a relatively small number of receptors, typically a few hundred, enable the discrimination of at least tens of thousands of odors (13) remains a major challenge. Emerging research indicates that even minor alterations in the functionality of a single receptor can lead to notable perceptual consequences (14–16). Despite our understanding that the OR gene family displays a significant genetic and functional diversity, the complex nature of the olfactory code and our limited knowledge about specific OR-odorant pairings pose challenges in translating this variability into perceptual distinctions.

In order to decipher the combinatorial code of odours, a considerable effort has been devoted to understand the interactions between ORs and molecules. Since the pioneering work led by G.M. Shepherd, several databases and datasets containing genomic, proteomic and functional information on olfactory receptors have been created (17–23). However, majority of these platforms serve as repositories for genomic and proteomic data related to olfaction and only a few of these online resources offer a list of known interactions between olfactory receptors and molecules. In addition, only one single dataset contains non-responsive OR-molecule pairs and none of the existing databases gather crucial information on the bioassays, nor on the stereochemistry or concentration of the tested molecules. To address these limitations we propose Molecule to Olfactory Receptor (M2OR) database, which is the largest and most comprehensive database of OR-molecule experiments available (Table 1).

**Table 1.** Summary of the existing OR-molecule databases listed by creation year

Database	Pairs	Mols.	ORs	Species	Web interface	Non-responsive pairs	Bioassay description	Year
OdorDB	402	95	812	27	✓	✗	✗	2000
ODORactor	4223	3000	1608	2	✓	✗	✗	2011
OlfactionDB	400	85	83	2	✓	✗	✗	2012
Cong *et al.*	15 693	244	720	2	✗	✓	✗	2022
OlfactionBase	874	330	150	2	✓	✗	✗	2022
M2OR (this paper)	51 395	768	1246	11	✓	✓	✓	2023

Pairs corresponds to a number of distinct OR-molecule pairs in the database. Mols. and ORs are, respectively, numbers of unique molecules and protein sequences. Check mark and cross mark indicate a presence of a web interface, an inclusion of non-responsive pairs and details about the experimental procedure (bioassay description).

M2OR compiles experimental data from 42 scientific articles (12,22,24–63) and contains information about OR-molecule interactions including non-responsive experiments, details on experimental procedures, concentrations tested, as well as curated stereochemistry properties of the molecules. The web interface includes an intuitive search engine with advanced search options for fine-grained queries based on molecules, receptors or experimental details. Users can access raw experimental results or a curated aggregation of the experiments to OR-molecule pairs. Batch search can be used to obtain results in bulk using several standard molecule and receptor identifiers. This database offers an easy access to the results of 25 years of OR-molecule bioassays with an unprecedented level of detail which will facilitate both *in vitro* and *in silico* deorphanization.

## Comparison with previous work

During the last decades, efforts to understand how an OR recognises an odorant have generated an impressive amount of data. Table 1 compares the different databases which have been created to provide access to these results. To date, only OdorDB (19) and OlfactionBase (23) allow for web-searching for OR-molecule pairs. However, these two databases offer a limited access to experimental results (874 pairs are available in OlfactionBase and OdorDB lists 95 odorants which are known to activate ORs). Developed in early 2010s, OlfactionDB (20) and ODORactor (21) webservers are not maintained anymore and a recent work by Cong *et al.* (22) only provided a raw dataset in a CSV format without any webserver.

Except Cong *et al.* (22), none of the available data sources contain non-responsive OR-molecule pairs, which are an integral part of the combinatorial code of odorants. In addition, the existing databases do not gather information on bioassays used to assess the OR-molecule response, stereochemistry properties of the molecules, nor their concentrations. In contrast, the philosophy of M2OR is to provide all available information which is pertinent to study olfaction, such as non-responsive experiments, details on experimental procedures, concentrations tested, as well as curated stereochemistry properties of the molecules.

### New features

Olfactory perception is dependent on odorant concentration and changes in concentration can lead to different perception of hedonicity or olfactory quality (64). From a molecular point of view, concentration of a ligand has a considerable influence on the response of ORs. An increase in the ligand concentration results in a higher probability of OR activation, ultimately leading to an increase in cellular signaling. This way, a molecule will not induce any cellular response at low concentration, whereas it will become an agonist for a large subset of ORs when its concentration increases. Contrary to previous databases, M2OR includes either screening concentration or EC_50_ for all gathered experimental data. This allows users to analyse OR-molecule interaction beyond simple responsiveness. Additionally, a great deal of effort has been invested in providing stereochemistry of molecules. Indeed, certain ORs, such as OR1A1, have a different response to enantiomers (39), making the complete curation of stereochemistry an essential aspect of M2OR.

Functional studies of ORs have relied on a variety of bioassays, including the use of native olfactory sensory neurons (OSNs) and several different heterologous expression systems that have facilitated the deorphanisation of ORs. However, it is crucial to take into consideration the instances of assay-dependent bias when interpreting OR responses (65). A recent demonstration illustrates this phenomenon: new ligands for ORs were successfully identified in human prostate carcinoma cell lines (LNCaP), whereas they were not recognised when ORs were expressed in HEK293 cells (66). As a result, M2OR incorporates information about experimental procedures, enabling users to identify and filter experiments based on various assay metadata, such as cell lines or assay types.

These unique features significantly increase the overall value and information provided by M2OR. For example, the assay metadata can be used to estimate response confidence level in a subsequent applications such as training a machine learning model (67). Information such as concentration, cell line or assay type facilitate bioassay experimental design and it provides a necessary data to study the impact of different experimental procedures on the OR-molecule pair response.

## Database construction

### Data extraction and curation

**Figure 1.**
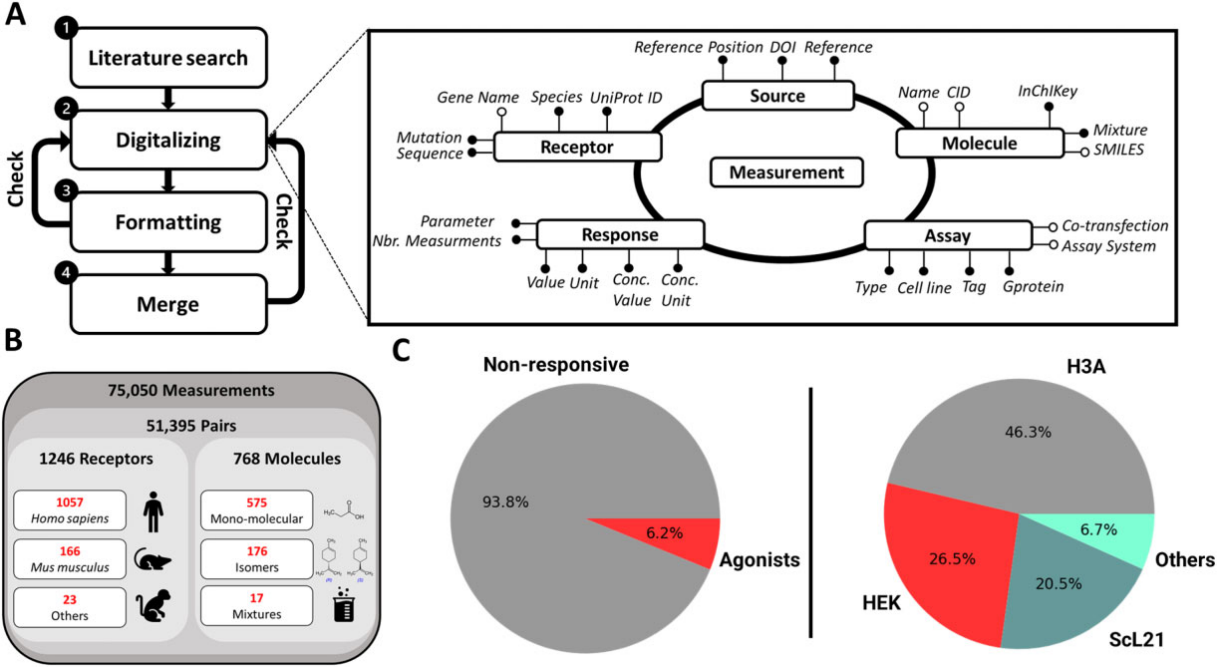
Summary of the construction and architecture of M2OR. (**A**) Database construction workflow: diagram shows the assembly process with framed figures for each category, subdivided further. Solid circles indicate mandatory columns and hollow circles are optional information. (**B**) Database overview of the distribution of data in terms of sequences and molecules. (**C**) Distribution of the number of agonists and non-agonists and the type of bioassay. H3A (Hana3a cell line) and Scl21 (stable cell line) are both derived from HEK (human embryonic kidney cell line).

A thorough and detailed workflow was employed to ensure the accuracy, consistency and reliability of the M2OR database. Firstly, a comprehensive list of scientific papers was collected using literature tools such as PubMed, Google Scholar and ResearchRabbit, and then sorted based on the estimated number of experiments. This approach allowed for the identification of valuable sources of data, which could be further curated to provide a robust and comprehensive database. To populate M2OR, the main text and supplementary material of each article are parsed through, and complementary information is, when needed, requested from the corresponding authors. This information is then carefully reviewed, checked and formatted to ensure its accuracy and consistency. A series of checks are also employed to highlight any errors such as typos or inconsistencies in the data (see Data consistency checks in Supporting material). This approach ensures that the data could be easily compared and analysed across different studies, thus providing a valuable resource for researchers interested in receptor-molecule interactions and their effects on the olfactory system. Figure 1A shows the pipeline followed for the construction of M2OR. Checking and formatting code as well as the raw information for each data source is freely available on M2OR GitHub repository (https://github.com/chemosim-lab/M2OR).

#### Molecular identifier

The International Chemistry Identifier Key (InChIKey) (68) serves as a standardized molecular identifier in the M2OR database. It is retrieved from PubChem (69) using the provided molecular identifiers found in the articles. Identifiers used by the authors were preferentially kept as stated in the orignial publication, and other identifiers, such as CID, were inferred from PubChem using InChIKey. Name and CAS are preferably retained as they are in the source, although there might be some inconsistencies due to CAS being a proprietary identifier and name not standardized. If only the molecular structure is available, a SMILES representation is inferred and further used to search for the corresponding InChIKey. In one study, the authors used a newly-synthesised molecule which is only identified by its SMILES string.

#### Compound classification

The diversity of the composition of the tested compounds is defined by three classes: ‘mixture’, ‘sum of isomers’ and ‘monomolecular’. The term ‘mixture’ indicates that the experiments were carried out using a blend of multiple molecules (e.g. essential oil or artificial composition) and mixtures are identified by a space-separated list of InChIKeys of each compound. For all the molecules, information on isomerism is carefully researched. Number of chiral centres and geometric isomerism are automatically determined from SMILES using the RDKit package. If a molecule has at least one chiral center and no specific information is provided about the enantiomery or the diasteroisomery, the molecule is considered to be a combination of isomers, and thus labelled ‘sum of isomers’. In cases where the authors explicitly mentioned isomerism of a tested molecule it is identified as ‘monomolecular’. All achiral molecules are also labeled as ‘monomolecular’.

#### Receptor identifier

Protein sequence serves as a standardized OR identifier in M2OR. If it is not available in the original publication, it is retrieved from UniProt (70) using the name or other identifier provided by the authors. For mutated receptors, the wild-type identifier and sequence is retrieved, and the mutation is separately indicated using *XpositionY* format where amino acid *X* from the wild-type sequence is mutated to amino acid *Y* at the given *position*. Additions are indicated by setting *X* to ‘+’ and deletion with ‘−’ as *Y*. The taxon name of the species from which the OR originates is also included.

#### Response

The results of the experiments are divided into two categories according to the authors conclusion. An experiment is labelled ‘responsive’ if the measured signal is considered as statistically different from the baseline by the authors. Otherwise it is labelled ‘non-responsive’. If available, different experimental parameters are collected such as the compound concentration, the assay raw response and EC_50_. Finally, if authors did not provide conclusion on the response (e.g. for screening experiments), a specific workflow is used to determine the response (see Mainland *et al.* (27) data treatment in Supporting material).

#### Bioassay

Exploring the recognition spectrum of ORs have required the development of several cell biology protocols to overcome the challenge associated with expressing ORs on the surface of heterologous systems cells (71). In the majority of OR assays, compounds are typically evaluated individually against each receptor using transient mammalian cell line-based luciferase assays. However, there are multiple methods to measure the molecule-induced activity of an OR and we have carefully extracted assay information from the publications. Briefly, we gather several parameters such as measured quantity, type of cell line for OR expression, type of G-protein, co-transfected protein or type of tags (i.e. N-terminal modifications). The experimental conditions and tools used are also included in the database.

#### Resources

The original source of data, including the reference and DOI, is collected. Additionally, a specific location of the information, such as table, figure or supplementary material is included in the database.

### Web implementation

M2OR is a dynamic web application developed using the Laravel framework (https://laravel.com/docs/10.x), which utilizes HTML, CSS, JavaScript and PHP for development. M2OR offers a user-friendly and interactive experience, enabling seamless data exploration and downloads. Additionally, a Content Management System (CMS) has been developed also on the Laravel framework to facilitate efficient management, editing, addition and deletion of data from the administration part. M2OR is powered by MySQL relational database management system (https://www.mysql.com/).

## Data content

M2OR contains 1246 unique protein sequences across 11 different mammalian species. *Homo sapiens* and *Mus musculus* ORs represent most of the experiments, with respectively 66% and 33% of the data (Figure 1B). The database includes 768 compounds, where 176 are sum of isomers, 407 are non-stereoisomeric molecules, and 168 are specific isomers. Moreover, 17 mixtures are also included in the database. Combination of these receptors and molecules results in a total of 51 395 unique pairs. With some pairs tested multiple times, the database contains 75 050 different experiments and represents the largest database available in the literature on OR bioassays (Table 1). With 3100 active pairs and, 48 295 non-responsive pairs, M2OR comprises 6% of agonist (Figure 1C). 41% of the bioassay results are luciferase assays using the Hana3A cell line, which expresses chaperon proteins like RTP1 or RTP2, olfactory G-protein and rho tag. This collection gives an overview of what has been achieved in terms of ligand binding assays since 1999.

### Experiments table

**Figure 2.**
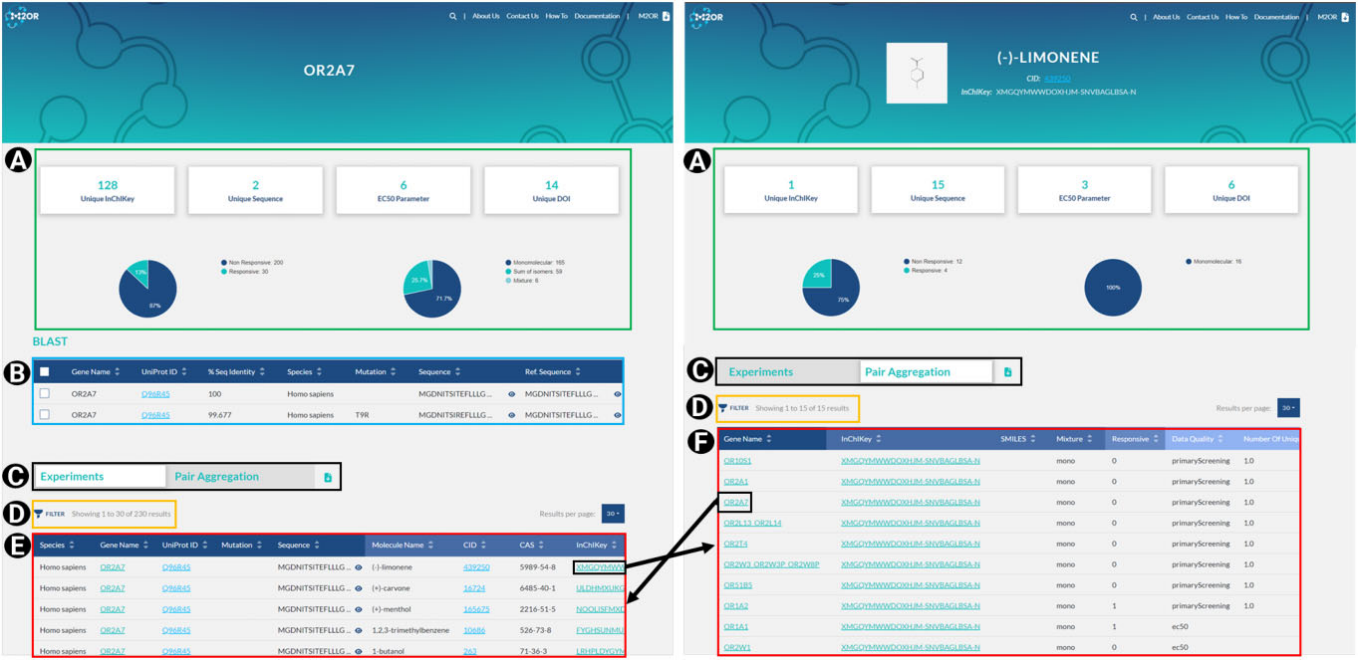
Search results. Receptor page (left) and Molecule page (right). They are subdivided as follows: (**A**) statistics for the displayed table, from left to right the number of unique molecules (Unique InChIKey), number of unique sequence (Unique Sequence), number of dose-response experiments (EC_50_ Parameter), number of associated references (Unique DOI). Pie chart on the left represents the ratio of responsive/non-responsive records for the displayed table. Pie chart on the right shows the ratio between sum of isomers, monomolecular compounds and mixtures. (**B**) BLAST table in the case of a receptor search. User can select one or more sequences related to the initial search. (**C**) Button to switch between Experiments table and Pair aggregation table. (**D**) Button to select filters. (**E**) Experiments table corresponding to the search query. Gene name and InChIKey link to Experiments table for a given receptor and a given molecule, respectively. UniProt ID and CID redirect the user to the corresponding record on UniProt and PubChem. (**F**) Pair aggregation table corresponding to the search query.

When searching in M2OR, the user is navigated to Experiments table which contains all relevant bioassay data. It includes several experiments per OR-molecule pair depending on the number of different types of experimental procedures performed on the pair. Molecules and receptors in Experiments table are respectively described by *Name*, *CID*, *CAS*, *InChIKey*, *SMILES* and *Mixture* for molecules, and *Gene name*, *UniProt ID*, *Sequence*, *Mutation* and *Species* for receptors (Figure 2D).

Details about the response are provided via columns: *Parameter*, *Responsive*, *Value*, *Unit*, *Concentration*, *Concentration unit* and *Nbr. measurements*. Responses are binary-coded in the *Responsive* column with 0 representing a non-agonist and 1 an agonist. The *Concentration* and *Concentration unit* columns contain either a concentration and its units, in the case of a screening experiment, or a maximum concentration and its units used in a dose-response assay (i.e. experiments performed at several concentrations). When a mixture of compounds was used for the experiment, the entry in the *Concentration* column is a comma-separated list of concentrations for each individual component. For screening experiments, the *Value* and *Unit* columns contain raw value reported in the source article, and for dose-response assays, the EC_50_ value and its units are reported in these columns. Which type of value is reported in the *Value* column is described by *Parameter*, which can be EC_50_ for dose–response measurements, Raw if the raw response is available or norm_rec, norm_pair, or norm_other for responses normalized by the receptor’s baseline, the response of a given pair, or an unknown normalization denominator, respectively. Additionally, number of repetitions is reported in the *Nbr. measurement* column.

Bioassays are fully described in the following columns: *Type*, *Cell line*, *Gprotein*, *Co-transfection*, *Tag*, *Delivery*, *Assay* and *Assay system*. Each bioassay is described by the measured quantity (*Type*), which could be a concentration of cAMP (CAMP), Ca^2+^, or Secreted embryonic alkaline phosphatase (SEAP), the fluorescence emitted by Green fluorescent protein (GFP) or by luciferase, or the membrane activity measured by intensity (Intensity) or conductance (Conductance). *Cell line* used for OR expression is also specified. This could be HEK293T cells (HEK), engineered OR-specific derivatives like Hana3A cells (H3A) and ScL21 (SCL21), yeast-based systems, oocytes, olfactory sensory neurons (OSN), Neuroblastoma x Glioma hybrid (NxG108CC15), or human cancer cells (HeLa/Olf). When available, the type of G-protein (*Gprotein*) is detailed (G_olf_, G_alpha16_, G_alpha15/16_, G_alpha q_). Any protein co-transfected with the ORs, as mentioned in the source, is listed in the *Co-transfection* column. N-terminal modifications, known as tags (Il-6-Halotag®, Flag, Rho, GFP, MYC, Rho Lucy), which can influence the response, are frequently used in these bioassays and are reported in the *Tag* column. Experimental conditions, such as *Assay* (*in vitro*, *ex vivo*) or *Delivery* method (liquid, gas), are also noted. The tools used, such as FLIPR or Glosensor, are mentioned in the *Assay system* column.

Finally, resources are indicated in the columns: *Reference*, *DOI* and *Reference Position*. The original source of the experiments, including the reference and DOI, is provided. Additionally, a specific location of the information, such as table, figure or supplementary material is noted in the *Reference position* column.

### BLAST table

Human genome includes approximately 1000 OR genes, of which around 60% are considered pseudogenes (7,72). Each OR has a distinct recognition spectrum and alterations in one or more amino acids can significantly change its response (73,74). Multiple variants and mutants of the same gene have been tested in the literature and they are gathered in the M2OR database. For instance, 42 different sequences share more than 99% sequence identity with the sequence annotated as OR1A1 in the UniProt database (70) (i.e. with the reference sequence) and some of these sequences show different responses compared to the reference. To facilitate the comparison of such cases, similar sequences are grouped under the same reference sequence. BLAST algorithm (75) is used to compare each sequence in M2OR against the UniProt database (2023 release 03). Name and UniProt ID of the best match in terms of identity are then associated with these sequences. They are subsequently grouped by their best match name, resulting in the BLAST table (Figure 2B). The BLAST table includes the name of the best match (*Gene name*), its UniProt ID (*UniProt ID*), its UniProt sequence (*Ref. sequence*), the query sequence (*Sequence*), an identity ratio between the query sequence and the sequence of the best match (*Seq. indentity*), the species of the receptor (*Species*) and the mutations required to transit from the Ref. sequence to the query sequence (*Mutation*).

### Pair aggregation table

Multiple experiments for the same OR-molecule pair can be found in Experiments table (e.g. multiple concentrations tested or different experimental procedures). However, users are often interested in an aggregated view on the OR-molecule pairs for applications such as analysis of the combinatorial code, or new active pair prediction (22,67,76,77). Pair aggregation table is created to provide this consensus response for each OR-molecule pair. Creation of this table relies on the following decisions:

1. Dose–response measurements are prioritized over screening data. When there are contradictory responses between multiple dose-response measurements for a given pair, then the pair is discarded. So far, this resulted in elimination of 27 pairs.
2. In case of the screening data, we exclude pairs that are responsive at low concentrations but not at higher concentrations. We also discard pairs that exhibit inconsistencies at the same concentration. This leads to elimination of additional 246 pairs.
3. A consistent screening pair is considered responsive if it is responsive in at least one concentration.

By applying these rigorous and systematic criteria, we obtain a set of 51 395 consistent OR–molecule pairs.

Pair aggregation table (Figure 2F) comprises *Responsive*, *Data quality* and *Number unique value screen* columns. In addition, there are the molecule and receptor information columns as in Experiments table. The *Responsive* column contains the consensus response of the pair and the *Data quality* column indicates reliability of the response decision. The most reliable are dose-response experiments (EC_50_). If only a screening was done, then secondary screening (secondaryScreening), with mediocre reliability, indicates that the pair was tested in at least two distinct concentrations and the least reliable primary screening pairs (primaryScreening) were tested in a single concentration. In case of a screening-based decision, *Number unique value screen* shows the number of distinct concentrations tested for a given pair.

### Navigating through M2OR

M2OR can be navigated via a web interface using four options:

1. Basic search: It enables the user to search for specific molecules or receptors using identifiers such as InChIKey, CID, SMILES, molecule name, receptor sequence, receptor name or UniProt ID. The results are displayed on a detailed ‘Listing’ page.
2. Advanced search: This feature allows the user to conduct detailed searches within the M2OR database using dropdown menu options or auxiliary search queries for each column. Boolean operators AND, OR and NOT are available for more granular control over search queries.
3. Batch search: With this functionality, the user can execute multiple search queries simultaneously by inputting identifiers such as sequences, UniProt IDs, Gene names, InChIKeys, CIDs or SMILES. The user can combine search criteria using boolean operators AND, OR and NOT.
4. Browsing table: All available experiments in the M2OR database can be browsed through Browsing table without any specific search.

#### Basic search

**Figure 3.**
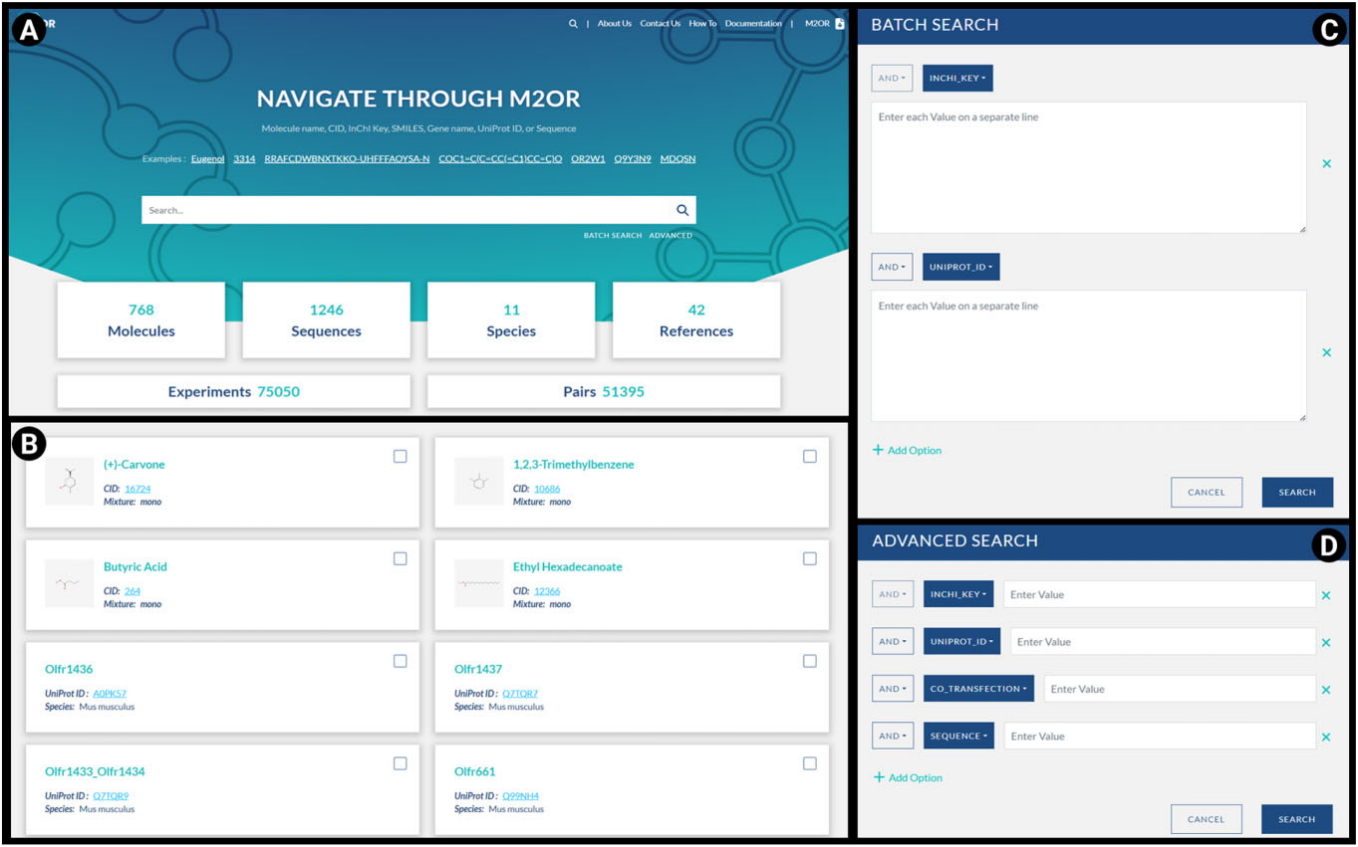
Search options within M2OR. (**A**) M2OR front page displaying the statistics and examples for Basic search. (**B**) Listing page resulting from the basic search. User can select several molecules or receptors and get access to the experimental results. (**C**) Batch search. The database can be explored by providing list of molecules (identified by name, CID, InChIkey or SMILES) or receptors (identified by sequence, gene name or UniProt ID). (**D**) Advanced search. Detailed queries can be executed within the M2OR database based on molecule, receptor, type of bioassays or reference.

The user can conduct a search for either molecules or ORs using the search bar located on the home page (Figure 3A). For molecules, InChIKey, CID, SMILES or common names can be entered. For ORs, a complete sequence, gene name or UniProt ID may be an input. Additionally, ORs can be identified using a segment of the sequence, such as specific motifs. After initiating search, the user will be directed to a ‘Listing’ page, which compiles all the records that correspond to the search criteria.

#### Listing

In the Listing page, each tile represents either a molecule or an OR and includes their main identifiers (Figure 3B). For molecules, these are a common name, a clickable CID that redirects to the corresponding PubChem page, and an InChIKey identifier. For receptors, the identifiers are the gene name, a clickable UniProt ID that redirects to the corresponding UniProt page, and the associated species.

The number of search results is displayed at the bottom left of the listing page, with additional pages available for navigation if the number of matches exceeds 30. The user can select a single tile or multiple tiles using a clickable box in the top right corner of each tile. Once the selection is finalized, the user can navigate to the corresponding Experiments table (see Experiments table section for further details). If a single tile was selected, the user is directed to *Molecule page* or *Receptor page*, depending on the choice. Otherwise a general *Experiment page* is shown.

#### Experiment/Molecule/Receptor page

Statistics pertaining to the displayed Experiments table are shown on top of the *Experiment page* (Figure 2A). There are four sections that respectively show the number of displayed unique molecules, unique receptors, EC_50_ experiments and sources linked to the table. Two pie charts offer a swift overview of the proportions between agonists and non-agonists and monomolecular compounds, sum of isomers and mixtures.

The table can be downloaded by clicking on the download icon. Above the table, the total number of records is shown, and the user can choose number of rows to be displayed per page. Each column can be sorted in ascending or descending order for ease of navigation. Filter (Figure 2D) is available for users to select a specific value for certain columns. For example, to get all known agonists with their corresponding EC_50_ values, the user can search for a given receptor of interest, and then via the Filter function select only responsive records obtained in a dose-response experiments. The statistics on top of the page are updated according to the selected filters.

Cells in the columns such as the *UniProt ID*, *CID* and *DOI* are clickable and link directly to the relevant UniProt, PubChem and reference web pages, respectively. *InChIKey* and *Gene Name* redirect to Experiments table corresponding to the desired molecule or receptor. Additionally, an ‘eye’ icon is available for users to view the full content of each cell. For *Molecule page* and *Receptor page*, the user can find a banner which provides information about the search query, similar to the details available on the tile in the listing page.

The user will also find a supplementary BLAST table in the *Receptor page*. This table enables selection of variants or mutants associated with the receptor by using the *Check box* column. The user can select multiple entries from this column, and conveniently select or deselect all entries with a single click at the top of the column. A complete description is provided in the BLAST table section.

#### Batch search

Batch search is a feature that allows users to execute multiple search queries simultaneously (Figure 3C). For instance, the user can search for responses of multiple receptors by inputting a set of sequences, each separated by a line. Batch search can be used for receptors using various identifiers, namely the full sequence, UniProt ID or receptor name. The same applies to molecules, where the corresponding InChIKey, CID or SMILES can be used as inputs. Furthermore, Batch search feature supports use of multiple criteria, and the user can manage their combination using boolean operators such as AND, OR or NOT.

#### Advanced search

Advanced search allows the user to execute detailed queries within the M2OR database (Figure 3D). Each row offers two options for input: the user can either manually enter a specific value or choose from the available options in a dropdown menu. Moreover, Advanced search function provides granular control over the search query through boolean operators AND, OR or NOT.

#### Browsing table

Browsing table can be accessed through the ‘Experiments’ tile at the main page. All the records in the M2OR database can be browsed from here. Columns can be sorted and the number of displayed records adjusted.

## Future development

Moving forward, M2OR aims to continuously incorporate new bioassay results. The database will be expanded to include additional content such as antagonists and inverse agonists, a list of orphan ORs, and details of genes and their chromosomal positions. The perceptual dimension of odours will also be taken into account by adding information on the odour of molecules or groups of molecules.

To improve the user experience and ease of exploration, we are working on introducing new features for navigating receptor sequences. This will involve an implementation of Ballesteros Weinstein numbering (78) allowing users to compare the influence of point mutations across different ORs. In addition, a snake-plot representation will be included to visualise important features along receptor sequences.

The main planned update is an integration of a web server based on our previously developed predictive model (67) which will provide users with a complete hypothetical combinatorial code for a given molecule. This will offer a valuable insight about interactions between molecules and receptors.

With these advances, M2OR will strive to remain a leading and comprehensive resource for scientists as well as enthusiasts exploring the fascinating world of olfactory receptors and their interaction with various molecules.

## Conclusion

We design Molecule to Olfactory Receptor (M2OR) database, which brings together 75 050 bioassay experiments for 51 395 distinct OR-molecule pairs. It provides a user-friendly and intuitive interface to allow for fast and easy navigation through the current state of the research on OR-molecule interaction. Users can obtain information on the activity of a chosen molecule or a group of molecules, or search for ligands for a specific OR or a group of ORs. Advanced search allows for fine-grained queries using various metadata such as species or experimental assay system, and the database can be queried by multiple inputs via a batch search. Finally, for a given search query, users can access and download a curated aggregation of the experimental data into a binarized combinatorial code of olfaction. Having an unprecedented level of details about molecules, receptors and their interaction, M2OR provides an essential tool to facilitate the exploration of the response spectrum of ORs. This database will provide a hub for researchers in olfaction and GPCR research.

## Supplementary Material

gkad886_Supplemental_File

## Data Availability

M2OR is freely available at https://m2or.chemsensim.fr/. Raw data and source codes are accessible at https://github.com/chemosim-lab/M2OR and https://doi.org/10.5281/zenodo.8385484.
