# Supplementary material for: M2OR: a database of olfactory receptor–odorant pairs for understanding the molecular mechanisms of olfaction
Source: Nucleic Acids Res. 2023 Oct 23;52(D1):D1370–9. doi: 10.1093/nar/gkad886 (PMC10767820; doi:10.1093/nar/gkad886)
Supplement: gkad886_Supplemental_File [file gkad886_supplemental_file.pdf]

Published online ?? MONTH ???? *Nucleic Acids Research, 2023, Vol. ??, No. ??* **1–2**
doi:10.1093/nar/gkn000

M2OR: A Database of Olfactory Receptor-Odorant Pairs for Understanding the Molecular Mechanisms of Olfaction

Maxence Lalis [1,†], **Matej Hladiš** [1,†], **Samar Abi Khalil** [1], **Loïc Briand** [2], **Sébastien Fiorucci** [1], **Jérémie Topin** [1,*]

[1]Institut de Chimie de Nice, Université Côte d'Azur, UMR 7272 CNRS, 06108 Nice, France and [2]Centre des Sciences du Goût et de l'Alimentation, CNRS, INRAE, Institut Agro, Université de Bourgogne, F-21000 Dijon, France

Received YYYY-MM-DD; Revised YYYY-MM-DD; Accepted YYYY-MM-DD

SUPPORTING MATERIALS

Data consistency checks

To ensure data accuracy and uniformity of the M2OR database, a comprehensive series of checks are performed to identify and correct errors or inconsistencies.

1. **No empty cells**: Columns *Parameter*, *Responsive*, *DOI*, *Sequence*, *Nbr. measurements*, *Type*, *Assay*, *Delivery*, *Cell line*, and *SMILES* need to be populated and *InChiKey* is populated unless the compound is custom-synthesized and its InChIKey is not available on PubChem. Additionally, when *Parameter* is EC_{50}, the *Value* and *Unit* columns must contain data.

2. **Molecule consistency**: SMILES format is validated to confirm it represents a single molecule and not a mixture. InChIKey format is checked and its presence in the PubChem database is confirmed. Molecule's stereochemistry is compared to the *Mixture* column; those with at least one undefined stereocenter within the SMILES representation should be labelled as "sum of isomers" and "mono" otherwise. If the record indicates a mixture, InChIKey should contain multiple space-separated values.

3. **Receptor Consistency**: Protein sequences can only contain 20 proteinogenic amino acids and be of an acceptable length (200-380). Sequence mutations are checked such that the amino acids at a given mutation position exist. Furthermore, a cross-verification process is employed between mutated sequences to identify and check for sequence and mutation combinations leading to identical mutants. The *Mutation* column format is further inspected.

4. **Response consistency**: EC_{50} values in a non-log units should either exceed 0 or be marked as "n.d." For the same data resource, identical OR-molecule pairs should produce consistent responses at equal concentrations. All columns corresponding to a bioassay must be filled with predefined values.

The entire database must pass these checks to qualify for updates. To ensure uniform formatting across records, automatic formatting operations are performed, which include white space normalization and deletion, case adjustments, and common typo rectification. An optional, more stringent checker is available to flag cases where manual curation may be required. This includes confirming if parsed names for a molecule are present among all names associated with a given InChIKey, and verifying stereochemistry information (cis/trans or R/S) in names to confirm the "sum of isomers" entry. Lastly, receptor sequences with length that do not fall between 300 and 330 are flagged and manually curated.

Mainland *et al.* data treatment

Mainland *et al.* (1) employed a screening procedure for testing 73 odorants against a library of 511 human olfactory receptors. The screening procedure involves three stages: primary, secondary, and dose-response measurements. In the primary screening, each odorant with one concentration and one injection is tested against each receptor to identify pairs that elicit a response. In the secondary screening, the top 5% of odorant-receptor pairs from the primary screening are tested in triplicates in a three different concentrations against a no-odor control. Finally, in the dose-response measurement, dose-response curves are constructed for odorant-receptor pairs that were significantly different from the no-odor baseline in the secondary screening. However, for the primary screening, the authors do not explicitly state the responsiveness for all the pairs. Therefore, we established the following procedure to conclude the responsiveness of these experiments:

*To whom correspondence should be addressed. Tel: +33 (0)4 89 15 01 32; Fax: +33 (0)4 92 07 61 51; Email: jeremie.topin@univ-cotedazur.fr
† The authors wish it to be known that, in their opinion, the first two authors should be regarded as Joint First Authors.

© 2023 The Author(s)
This is an Open Access article distributed under the terms of the Creative Commons Attribution Non-Commercial License (http://creativecommons.org/licenses/by-nc/2.0/uk/) which permits unrestricted non-commercial use, distribution, and reproduction in any medium, provided the original work is properly cited.

We normalized the raw luciferase response using Z-score normalization

$$z(m,r,d) = \frac{(y(m,r,d) - B(r,d)) - \mu_{m,d}(y(m,r,d) - B(r,d))}{\sigma_{m,d}(y(m,r,d) - B(r,d))} \quad \textbf{(1)}$$

where $y(m,r,d)$ is the raw response at a day d for a molecule m and a receptor r, $B(r,d)$ corresponds to a basal activity of the receptor r at a day d, and $\mu_{m,d}(.)$ and $\sigma_{m,d}(.)$ are, respectively, mean and standard deviation for all experiments for a given receptor r (i.e. through all tested molecules and all days). An experiment is considered responsive if the normalized response is greater than a decision threshold k:

$$\frac{1}{D}\sum_{d=1}^{D} z(m,r,d) \geq k \quad \textbf{(2)}$$

where k is selected to maximize the sensitivity/specificity ratio of the Receiver Operating Characteristic curve (ROC). Several normalization methods were considered and Z-score has the highest area under the ROC curve (AUROC) when a dose-response measurement is considered to be a true label and primary screening is a predictor (data not shown).

REFERENCES

1. Mainland, J. D., Li, Y. R., Zhou, T., Liu, W. L. L., and Matsunami, H. (2015) Human olfactory receptor responses to odorants. *Scientific data,* **2**(1), 1–9.
